# Evaluation of two rotational helmet technologies to decrease peak rotational acceleration in cycling helmets

**DOI:** 10.1038/s41598-022-11559-0

**Published:** 2022-05-11

**Authors:** Thomas Hoshizaki, Andrew M. Post, Carlos E. Zerpa, Elizabeth Legace, T. Blaine Hoshizaki, Michael D. Gilchrist

**Affiliations:** 1 Department of Kinesiology, University of Waterloo, 200 University Avenue West, Waterloo, ON N2L 3G1 Canada; 2 Department of Human Kinetics, University of Ottawa, Ottawa, Canada; 3 School of Kinesiology, Lakehead University, Thunder Bay, Canada; 4 School of Mechanical and Materials Engineering, University College Dublin, Dublin, Republic of Ireland

**Keywords:** Biomedical engineering, Mechanical engineering, Materials for devices, Structural materials, Risk factors

## Abstract

The risk of brain trauma has been associated with the rotational kinematics leading to the development of helmets with a variety rotational management technologies. The purpose of this paper was to employ a rotation specific test protocol to evaluate the effectiveness of two of these technologies. Dynamic response of the head was measured to assess the performance of each technology. Three cycling helmets with identical construction were included in this study. One helmet with no rotational technology, an established, commercial technology and a novel helmet rotational technology designed and assembled by the authors were tested. A drop test onto a 45° anvil was used to measure the ability of each helmet to manage the dynamic response of the head form during a series of impacts. The results revealed both rotational helmet technologies resulted in lower peak rotational acceleration and brain strain, however each technology demonstrated unique performance characteristics depending on the impact condition.

## Introduction

The health care system in the United States requires two billion dollars a year to treat and manage head injuries in the general population^1,2^. Accounting for 30% to 40% of hospitalizations for children and adolescents^3^, concussion is the most common cause for head injuries in children while cycling. Sport-related concussions in general have been described as a “silent epidemic”, with the prevalence and effects of these injuries not fully described^1–5^. Helmet technologies have been developed to minimize the risk head trauma including concussions from head impacts in sport^5–8^. While they have proven useful in reducing head injuries in cycling, the mechanism of injury associated with concussion is not fully reflected in current helmet testing protocols^9–12^. Rotational kinematics describing head dynamics during impacts has been associated with diffuse brain injuries including concussion^9–11,13–17^. However, the effectiveness of sport helmets to manage head trauma typically involves measuring linear acceleration using a vertical drop test to a flat anvil^18^. Post et al.^19^ and Rowson et al.^20^ reported a weak relationship between peak rotational and linear accelerations for head impacts. While several test methods have been employed, to date no cycling helmet test standard includes a rotational performance criterion^10,12,14,21–27^. A proposed high friction impact test protocol was used to measure a helmets capacity to manage rotational acceleration. It included a free drop helmeted head form to an angled impacting anvil fitted with high friction sandpaper.

Cycling helmet manufacturers increased interest in integrating effective rotational management technologies into cycling helmets followed reports that rotational impact characteristics are important predictors of risk for concussive injuries^6,10–12^.

The purpose of this study was to evaluate the capacity of two rotational helmet technologies fitted to the same make and model of cycling helmet to manage impact trauma using a high friction impact test protocol.

## Methods

### Cycling helmets

**Figure 1:**
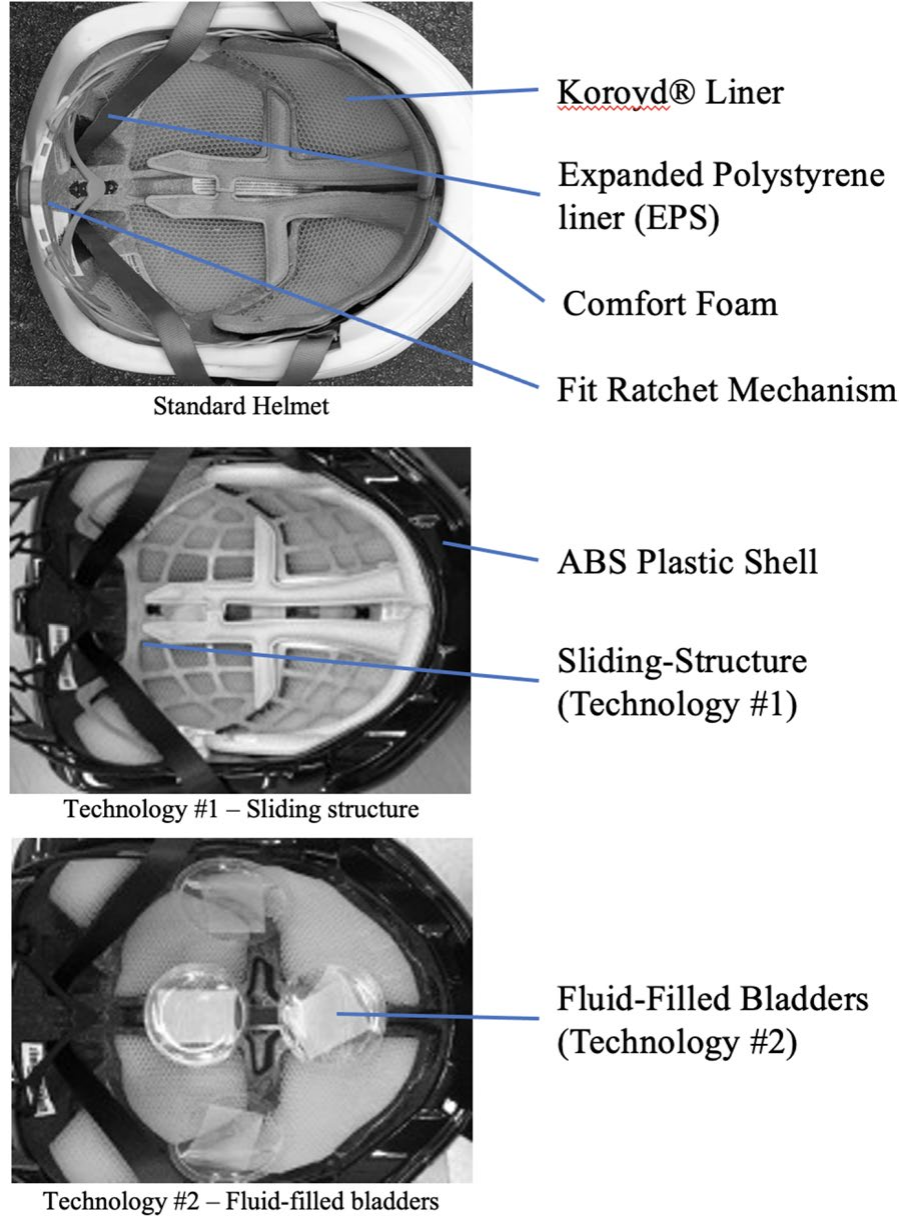
Cycling helmets tested, standard helmet (top), Technology #1 (MIPS), (centre), and Technology #2, (bottom).

Three identical medium sized cycling helmets were included in this study: a commercially available cycling helmet with no rotational technology, a commercially available cycling helmet with rotational technology low-friction layer (MIPS, Multi-Directional Impact Protection System, Sweden), and a commercially available cycling helmet fitted with a novel rotational technology composed of fluid filled bladders (Fig. 1). All three helmets were composed of a polycarbonate micro shell, expanded polystyrene liner, Koroyd^®^ inserts, fit pads, and a chinstrap. The sample size included four helmets for each helmet type for a total of twelve helmets.

### Rotational technologies

A number of rotational mitigation technologies have been introduced to the market including Wavecell a copolymer structure, SPIN a foam gel material, Leatt 360° turbine, 6D ODS suspension technology and Atomic AMID with a compression—shear material. The technologies used in this research included the industry leader Multi-Directional Impact Protection System, MIPS system and a novel fluid filled bladder technology. For this research helmet 1 (Standard Liner) was a standard commercially available helmet with no rotational management technology. Helmet 2, identified as Technology #1 was a commercially available cycling helmet with a rotational acceleration management system (Multi-Directional Impact Protection System, MIPS) and Helmet 3 identified as Technology #2 had the fit foam replaced three thermoplastic urethane (TPU) bladders containing low-viscosity fluid arranged to produce a low friction response between the head and the liner^28,29^. Each bladder was composed of TPU 85 plastic film, containing 3 ml of colorless mineral oil with an overall diameter of 65 mm and thickness of 3 mm. The thickness (3 mm) was the same as the fit foam they replaced to ensure proper fit. The helmets with rotational technologies did increase helmet mass slightly (25–45 g)^30^. Other than the rotational technologies all three helmets were identical in construction (Fig. 1).

### Equipment

**Figure 2:**
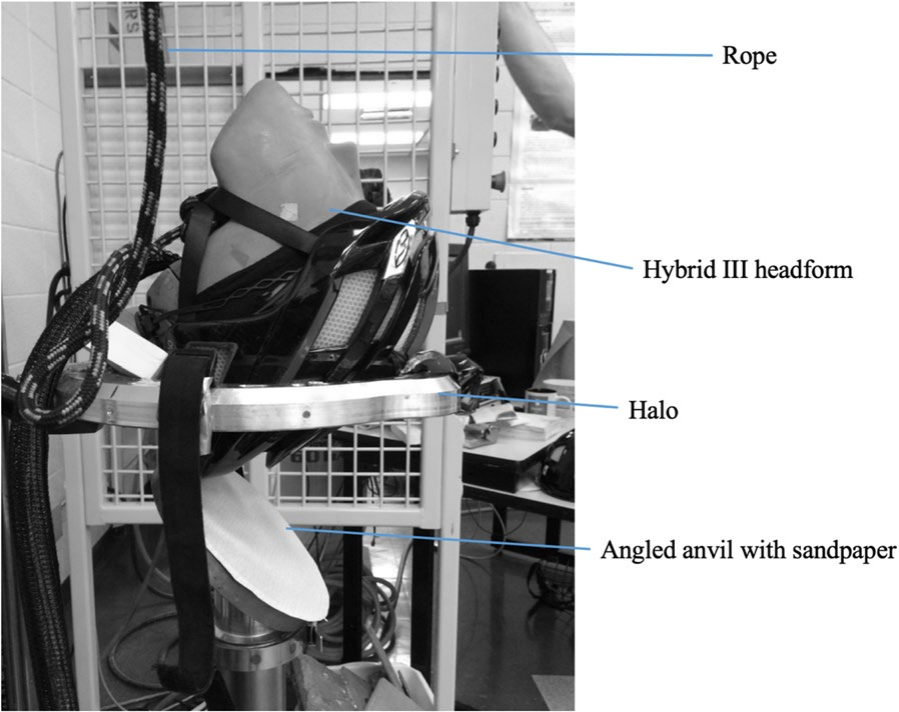
Drop rig, halo and 45° anvil with the sandpaper affixed.

A monorail drop rig system manufactured by Cadex Impact Inc. (St. Jean-sur-Richelieu, Quebec, Canada) as shown in Fig. 2 was used to impact the helmets. This apparatus was used in conjunction with a Hybrid III head form to perform a free drop. The halo supported the helmeted head form and passes around the anvil to allow an accurate impact to the headform location without interference from the travelling rig or anvil. An adjustable laser velocity measurement device was used to obtain the velocity of the headform just before impact. While several test methods that use neck forms are available, a free drop system was chosen for this research to ensure the effect of the neck form on the head form response was minimized^30–33^. The Hybrid III head form was used to include the most common 3-2-2-2 acceleration array^34,35^.

### Headform and sensor array

A 50th-percentile Hybrid III head form was placed on the monorail drop rig fitted with a free drop halo Fig. 2. The Hybrid III head form was fitted with nine-uniaxial Endevco accelerometers in a 3-2-2-2 array collecting at 20,000 Hz. The 3-2-2-2 Array was based on Padgaonkar et al.’s^35^ research to capture both rotational and linear accelerations. The accelerometers were connected to a DTS TDAS-Control (DTS, San Juan Capistrano, California, USA) control module used in impact simulations. The signals were processed using a CFC 1000 filter according to SAE J211 convention.

### Procedure

**Figure 3:**
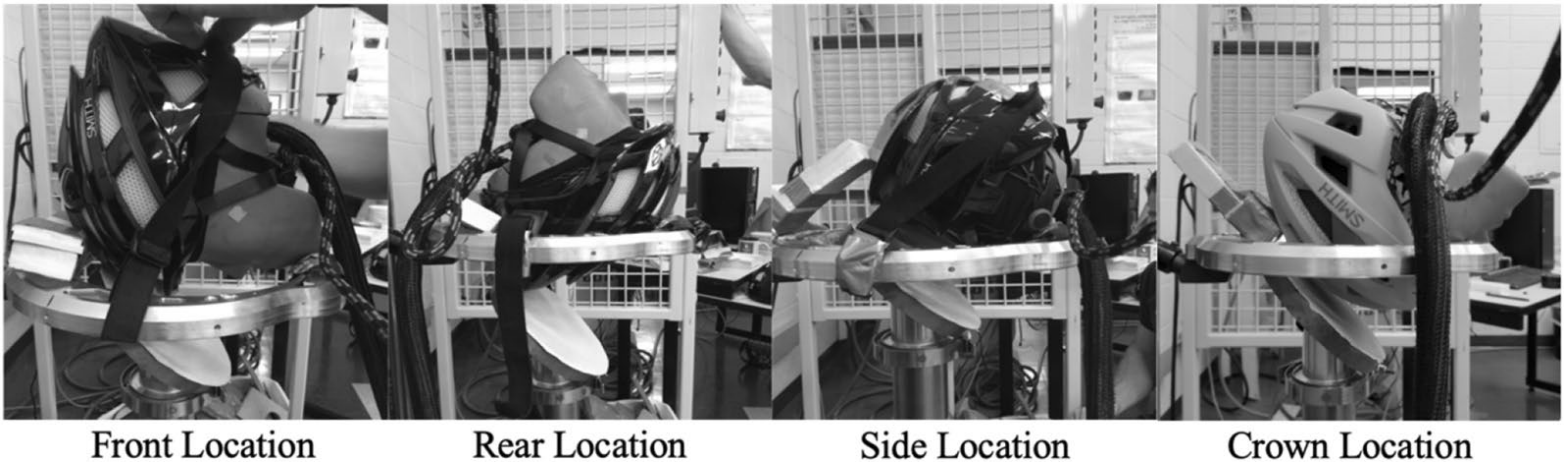
Headform placement for impacting the anvil in the front, rear (back), side and crown conditions.

The helmet was fixed to the head form using the retention strap and checked to ensure it was aligned to the same landmarks on the head form. The helmeted head form was placed on a halo attached to a monorail and dropped onto a 45° angled anvil with 80-grit sandpaper adhesive applied to the surface^9,36,37^. The impacting velocity was set at 6.5 m per second (SD ± 0.2). The impact locations included front (Rot Y axis), side (Rot X axis), crown (Rot Y axis), and rear (Rot Y axis) and defined relative to the angled impactor (Fig. 3).

### Finite element model

To obtain maximal principal strain (MPS) for cycling helmeted impacts, the linear and rotational loading curves for each impact were used as inputs into the University College Dublin Brain Trauma Model (UCDBTM)^38,39^. The model was developed using computed tomography (CT) and magnetic resonance imaging scans (MRI) of a male human cadaver to develop the head geometry of the UCDBTM^40^. The three-dimensional finite element model included the scalp, skull, pia, falx, tentorium, cerebrospinal fluid (CSF), grey and white matter, cerebellum and brain stem, represented by approximately 26,000 hexahedral elements^40^. The brain tissue was characterized as viscoelastic in shear with its brain behaviour represented by a linear viscoelastic model. The compressive nature of the brain tissue was defined as elastic. Shear characteristic of the viscoelastic brain was represented by the Eq. (1), where G1, is defined as the long-term shear modulus, G0, is the short-term shear modulus, and b is the decay factor^40^:

$${\text{G}}\left( {\text{t}} \right) = {\text{G}}_{\infty } + \left( {{\text{G}}_{0} - {\text{G}}_{\infty } } \right){\text{e}} - \upbeta {\text{t}}.$$

Validation of the UCDBTM was performed against cadaveric pressure responses and brain motion research^38,40^ Hardy et al.^39^. Comparisons of the model’s intracranial pressure response were also made to Nahum et al.^41^ experiments involving impacts with both rotational and linear acceleration components. The intracranial pressure response of the model was also found to be in good agreement with the cadaveric pressure responses of Hardy et al.^38^ with respect to general shape and duration Horgan and Gilchrist^40^. The brain motion traces of the model were found to be similar to research conducted by Trosseille^42^ Further examination of the model was performed using reconstructions of real-world brain injury incidents in which good agreement was found for magnitudes of brain stresses and strains compared to the literature. The peak strain values for the white matter and the grey matter are reported as the maximal principal for white matter and grey matter. It should be noted that the maximal principal strain values are included in this paper as a measure of brain trauma and do not represent levels of brain injury. For that reason, strain values were not included in the statistical analysis.

### Statistical analysis

A series of repeated-measures ANOVAs compared peak resultant linear and rotational acceleration for each helmet type. An ANOVA was completed for each of the impacted locations with comparisons of the effect for each helmet technological strategy to baseline and comparing each helmet technology. Pairwise comparisons (α = 0.05) were run for each metric, peak linear and rotational acceleration.

## Results

**Figure 4:**
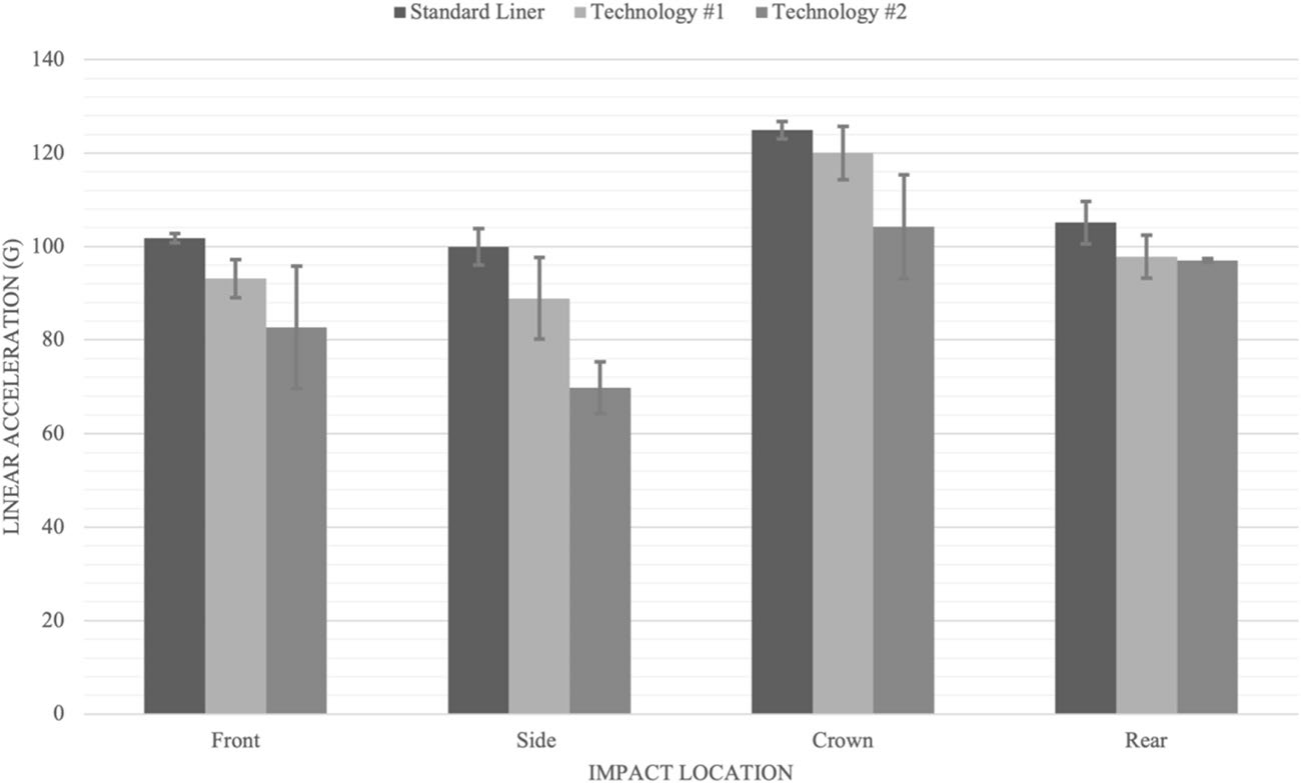
Bar graph displaying peak linear acceleration results in g for impact location and helmet type.

A statistical analysis was completed for the dynamic response (peak linear and rotational acceleration) variables. The maximal principal strain for grey and white matter were included in the results for reference only. For the front impact location pairwise comparisons for peak linear acceleration revealed no significant differences between the standard liner and technology #1 (p = 0.230), and technology #1 and technology #2 (p = 0.159). However, there was a significant reduction for peak linear acceleration between the standard liner and technology #2 (p = 0.026). For peak rotational acceleration there were significant differences between the standard helmet and technology #1 (p = 0.006), the standard helmet and technology #2 (p = 0.000), as well as between technology #1 and #2 (p = 0.000) (Table 1; Fig. 4).

**Table 1:** Summary table with means of impact variables measured with the standard deviations in brackets for the front location.

Front	Resultant acceleration		Brain tissue deformation	
	Linear (g)	Rotational (rad/s^2^)	White matter strain (MPS)	Grey matter strain (MPS)
Standard liner	101.8 (1)*	9266 (75)*	48.39 (5.03)	75.32 (1.29)
Technology #1	93.1 (4.1)	7919 (548)*^#^	42.19 (2.03)	69.04 (3.33)
Technology #2	82.7 (13.1)*	5310 (407)*^#^	33.04 (1.56)	59.88 (7.52)

*Indicates significant difference (alpha level p < 0.05), when the two technologies are compared to the standard liner.

^#^Indicates significant difference (alpha level p < 0.05), when the two technologies are compared to each other.

**Figure 5:**
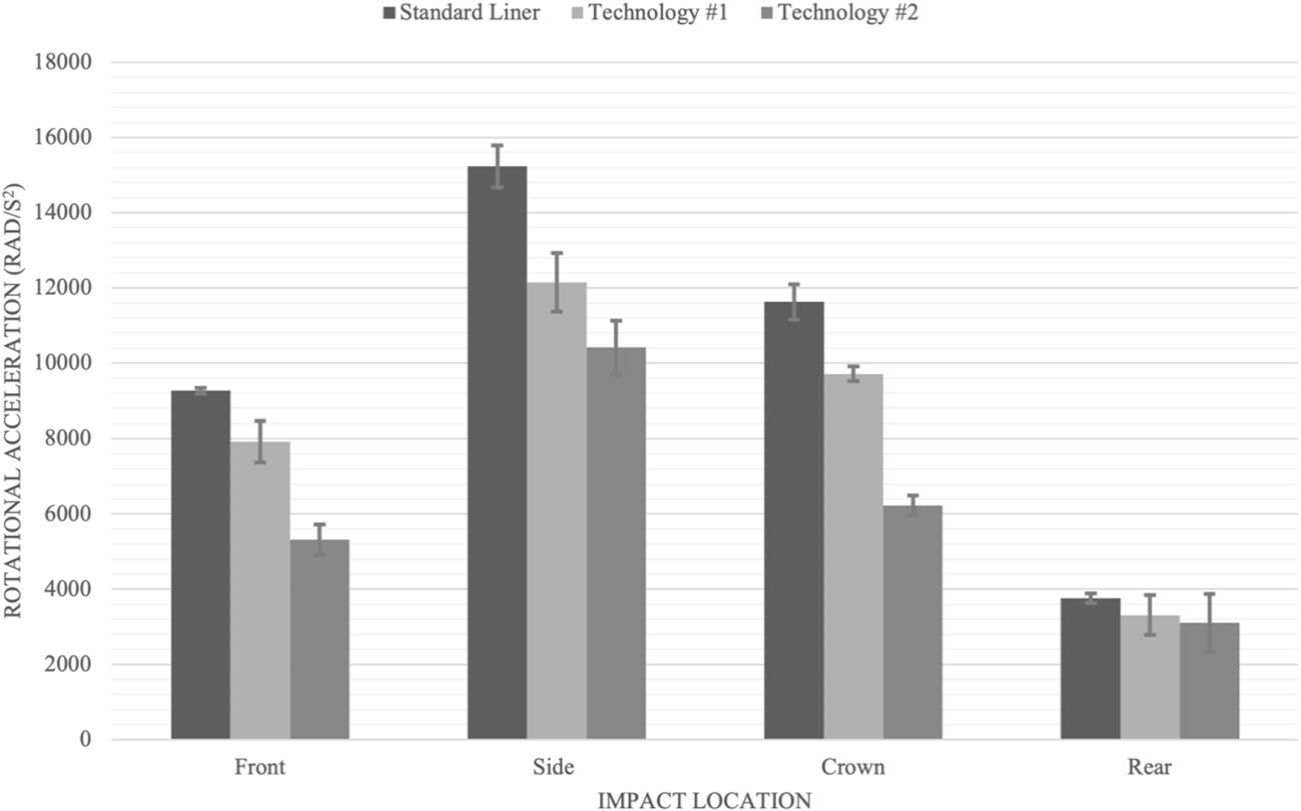
Bar graph displaying peak rotational acceleration results in radians per second squared (Rad/s^2^) for impact locations and helmet type.

For the side impact location, pairwise comparisons for peak resultant linear acceleration responses revealed no significance between the standard liner and technology #1 (p = 0.078) (Fig. 5). There were significant differences between the standard liner and technology #2 (p = 0.001) and between technology #1 and technology #2 (p = 0.010). For peak rotational acceleration pairwise comparisons revealed all three helmet types were significantly different from each other; the standard liner compared to technology #1 (p = 0.002), standard liner compared to technology #2 (p = 0.000) (Table 2).

**Table 2:** Summary table with means of impact variables measured along with the standard deviations in brackets for the side location.

Side	Resultant acceleration		Brain tissue deformation	
	Linear (g)	Rotational (rad/s^2^)	White matter strain (MPS)	Grey matter strain (MPS)
Standard liner	99.9 (3.9)*	15,227 * (554)	75.69 (3.02)	97.35 (2.90)
Technology #1	88.9 (8.7)^#^	12,149 (780)*^#^	67.95 (4.45)	81.46 (4.44)
Technology #2	69.8 (5.5)*^#^	10,412 (716)*^#^	63.54 (2.64)	75.43 (2.64)

*Indicates significant difference (alpha level p < 0.05), when the two technologies are compared to the standard liner.

^#^Indicates significant difference (alpha level p < 0.05), when the two technologies are compared to each other.

For the crown impact location pairwise comparisons revealed no significance for peak linear acceleration between the standard liner and technology #1 (p = 0.344) or between technologies #1 and #2 (p = 0.778). There was a significance difference between the standard liner and technology #2 (p = 0.034). For peak rotational acceleration pairwise comparisons revealed no significant difference between the standard liner and technology #1 (p = 0.344) and technologies #1 and #2 (p = 0.778) however there was significance between standard liner and technology #2 (p = 0.778) (Table 3; Fig. 5).

**Table 3:** Summary table with means of impact variables measured along with the standard deviations in brackets for the crown location.

Crown	Resultant acceleration		Brain tissue deformation	
	Linear (g)	Rotational (rad/s^2^)	White matter strain (MPS)	Grey matter strain (MPS)
Standard liner	124.9 (1.9)*	11,625.1* (473)	37.84 (1.95)	59.79 (3.04)
Technology #1	120 (5.7)	9714.8 (197.9)	27.57 (11.16)	47.84 (11.15)
Technology #2	104.2 (11.1)*	6227.5 (269)*	29.36 (0.45)	55.61 (0.450)

*Indicates significant difference (alpha level p < 0.05), when the two technologies are compared to the standard liner.

For the rear location, pairwise comparisons for peak linear and rotational accelerations between helmet types revealed no significant differences (Table 4; Figs. 4 and 5).

**Table 4:** Summary table with means of impact variables measured along with the standard deviations in brackets for the rear location.

Rear	Resultant acceleration		Brain tissue deformation	
	Linear (g)	Rotational (rad/s^2^)	White matter strain (MPS)	Grey matter strain (MPS)
Standard liner	105.1 (4.5)	3760 (129)	15.95 (0.72)	26.91 (1.09)
Technology #1	97.8 (4.6)	3308 (526)	20.41 (11.35)	35.05 (11.34)
Technology #2	97 (0.4)	3103 (774)	13.33 (1.85)	23.05 (1.85)

## Discussion

The testing protocol used in this research identified differences between the capacity of the three helmets tested in managing peak linear and rotational acceleration (Tables 1, 2, 3, 4). The low friction layer in technology #1 (MIPS) and fluid-filled bladders in technology #2 reduced the peak linear and rotational accelerations. While both technologies consistently outperformed the conventional helmet technology #2 was more effective in managing both linear and rotational accelerations for the front and side impacts. While technology #2 did significantly decrease linear and rotational acceleration for the crown impact site technology #1 did not perform as well (Fig. 5). Differences in the two technology structures likely contributed to the differences in the ability of the two technologies in managing linear and rotational accelerations for the four impact sites. Both technologies decreased peak linear and rotational accelerations at the rear impact site, however it was not statistically significant. This finding was not unexpected as both rotational technologies did not fully cover the rear impact site. As well, there was pronounced geometry at the rear impact site increasing the variance in the measurements thus decreasing the likelihood of statistical significance. The standard cycling helmet did not manage dynamic response during impacts as effectively as the two helmets with rotational technology. The two rotational technologies also resulted in decreased strain for both the grey and white matter when compared across all impact conditions (12.70–27.16%). The results demonstrated the two rotational technologies in this study were effective in decreasing peak linear and rotational acceleration for the conditions tested (Fig. 5). While no attempt was made to optimize the rotational technologies for specific impact conditions including impact location and direction these results support the notion that a specific technology may be more effective in managing rotational acceleration under specific impact conditions. Further research should consider investigating the opportunity to optimize helmet performance using to impact condition specific technology. The improved protection provided by rotational technology in this research supports the importance of including rotational test protocols in helmet certification standards.

## Limitations

As with all laboratory research not involving human participants the application and interpretation of these results must consider the limitations of the tools used to measure the impact trauma. The impacts used in this testing did not include a specific test to create acceleration around the Z axis. The association of acceleration in this axis with the risk for brain trauma supports adding an impact to create acceleration in the Z axis in future research. This research involved two unique technologies as examples of rotational management technologies and cannot be considered representative of other rotational technologies. The shell geometry of this helmet is considered aerodynamic; thus, the shape is not round. These conclusions are specific to cycling helmets tested and may be different for other helmets. The scope of the research was limited to head trauma in cycling^43–46^. Finally, the impacting protocol was a modification of existing methods proposed by the European test method for rotational impacts Halldin et al.^30,36,43^.

## Conclusions

The purpose of this work was to evaluate the effectiveness of a rotational specific test protocol in evaluating two technologies designed to decrease rotational accelerations during head impacts in cycling helmets. The findings revealed the rotational specific test demonstrated significant reductions for peak rotational acceleration for both rotational technologies when compared to the conventional helmet. Technology #1 had significant reductions in rotational acceleration for the front and side impact sites while Technology #2 had significant reductions in rotational acceleration for the front, side and crown impact sites. Each technology demonstrated unique performance characteristics depending on the impact condition. This research supports the use of helmet rotational test protocols to evaluate helmet capacity in managing the risk of head trauma.

## Supplementary Information

Supplementary Information.

## Data Availability

Raw and processed physical and computational data of all variables are currently being stored at the University of Ottawa, Canada. It is available upon request as well has been available in “supplementary files”.
